# Eating Competence among Brazilian Adults: A Comparison between before and during the COVID-19 Pandemic

**DOI:** 10.3390/foods10092001

**Published:** 2021-08-26

**Authors:** Fabiana Lopes Nalon de Queiroz, Eduardo Yoshio Nakano, Raquel Braz Assunção Botelho, Verônica Cortez Ginani, António Raposo, Renata Puppin Zandonadi

**Affiliations:** 1 Department of Nutrition, Faculty of Health Sciences, Campus Universitário Darcy Ribeiro, University of Brasília, Brasilia 70910-900, DF, Brazil; raquelbotelho@unb.br (R.B.A.B.); vcginani@gmail.com (V.C.G.); renatapz@unb.br (R.P.Z.); 2 Department of Statistics, University of Brasilia, Brasilia 70910-900, DF, Brazil; eynakano@gmail.com; 3 CBIOS (Research Center for Biosciences and Health Technologies), Universidade Lusófona de Humanidades e Tecnologias, Campo Grande 376, 1749-024 Lisboa, Portugal

**Keywords:** eating competence, eating behavior, COVID-19, diet, food choice

## Abstract

The coronavirus pandemic started a worldwide emergency, and tight preventive actions were necessary to protect the population, changing individuals’ daily habits. Dwelling and working at home can change dietary habits, affect food choice and access, as well as the practice of physical activity. In this regard, this study’s goal was to compare eating competence (EC) among Brazilian adults before and during the coronavirus pandemic, using the Brazilian version of the eating competence Satter inventory (ecSI2.0™BR) with the “retrospective post-then-pre” design. This cross-sectional study was performed from 30 April to 31 May 2021 among a convenience sample of the Brazilian adult population using an online platform (Google^®^ Forms). In the studied sample (*n* = 302 in which 76.82% were females), EC total score lowered during the pandemic (31.69 ± 8.26 vs. 29.99 ± 9.72; *p* < 0.005), and the decrease was worst after the beginning of the pandemic among those who reported weight gain, decreased the consumption of fruit and vegetables, and increased the consumption of sugary beverages. The contextual skill component seems relevant in this scenario, where our life and routines were changed entirely, demonstrating that the ability to manage the food context is essential, especially when sanitary and economic situations represent a new challenge.

## 1. Introduction

The pandemic of coronavirus started a worldwide state of emergency. Until 31 May, Brazil had registered 16,545,554 confirmed cases of COVID-19 and 462,791 deaths [1]. The measures to control and prevent the disease differed among Brazilian regions. However, the most common was social distancing to control individuals movability, like the schools’ and universities’ closure, as well as non-essential commerce, public areas, and the use facial masks [2]. While this strict preventive measure is essential to protect people, it can radically change individuals’ quotidian habits. Staying and working at home can affect diet, food choice, and access to food and limit the practice of physical activity [3].

Containment measures like self-isolation and social distancing substantially impact the population’s everyday life and a wide range of psychosocial impacts on individuals [4]. Stress factors like uncertainty about the duration of the quarantine, fear of possible infections, boredom, and fear of financial losses impact lifestyle and dietary habits [5]. For the general population, the lack of physical activity and poor mental health are the most critical risk factors for morbidity by chronic diseases. However, it is more worrisome for elderly and chronically ill patients at a higher risk of COVID-19-induced mortality [6].

Eating is complex and composed of learned behavior, social expectations, acquired tastes, attitudes, and feelings about eating and/or food items [7]. Eating competence (EC) is an attitudinal and behavioral view established by the Satter eating competence model (ecSatter). This concept is not centered on nutrients, portion size, or food groups, but on appreciating food and eating, with attention to the variability in the diet, paying attention to signals of hunger and satiety, and preparing nourishing meals with attention to the environment in which the food is consumed [7,8]. Four components compose the EC: eating attitudes; food acceptance; internal regulation; and contextual skills [7,8].

Considering eating behavior, home confinement leads to an altered food cue exposure, which can enhance impulsive eating behavior and emotional eating [9]. In a French study, the lockdown was associated to changes on food choice motives, remarkably increased mood as a food choice motive for 48% of the individuals [10]. Moreover, eliminating social eating practices could reduce mindful eating, which might negatively influence dietary choices and promote overeating [11]. Combining isolation and socioeconomic hardship with the deterioration of psychosocial health and a sedentary lifestyle might negatively affect eating behavior. Furthermore, the negative impact on metabolic health might worsen obesity and its metabolic comorbidities [9]. Regarding physical health, even a short period of overeating with abruptly reduced physical activity can have several consequences for health-related to a proinflammatory state [12]. It is associated with the development of metabolic syndrome leading to an increase in the risk of multiple chronic diseases and the development of the severe form of COVID-19 infection [9,12]. Nutrition becomes a priority during pandemic time, considering the situational stress-eating due to confinement [13].

Some studies evaluated the effect of lockdowns and restrictions connected to the COVID-19 pandemic on dietary–lifestyle behaviors shifts [3,6] and revealed substantial changes in dietary habits at the beginning of the pandemic. For example, in Poland, a significant percentage of individuals started eating and snacking more, leading to weight gain in previous overweight and obese subjects, aggravating this condition [14]. In Italy, a survey evaluating the connection between eating habits, mental and emotional mood showed that individuals tended to consume more comfort food and increase food intake to feel better [5]. On the other hand, Rodríguez-Pérez et al. found that Spanish adults have adopted healthier dietary behaviors closer to the Mediterranean diet during home confinement [15]. In Brazil, an increase in the ingestion of ultra-processed foods at the beginning of the pandemic was reported [16,17].

Given that the prolonged or intermittent social restriction may be needed until 2022 in some countries [18], it is essential to have the best possible understanding of its effects on people’s everyday life. Due to the restrictions related to the measurement confinement, it is hypothesized that, during the pandemic, there were changes in EC and food consumption compared to the period prior to the pandemic. In this sense, our study focused on comparing eating competence (EC) among a sample of Brazilian adults before and during the coronavirus pandemic, using the Brazilian translation of the eating competence Satter inventory (ecSI2.0™BR). It is expected that the findings of changes in EC and its four components may support the development of appropriate recommendations for lifestyle modifications during this time.

## 2. Materials and Methods

This cross-sectional study was performed from 30 April to 31 May 2021 among the Brazilian adult population using an online platform (Google^®^ Forms) accessible by any device with an Internet connection. The participants (convenience sample) were recruited using a link disseminated through social networks (WhatsApp, Instagram, Facebook, email list, and by researchers’ personal contact). The inclusion criteria were: being Brazilian adults (20–59 years old, according to the Brazilian Health Ministry [19]); agreed to participate after they were told about the purpose of the survey; and completely filled out the questionnaire.

We used a self-administered survey using online application due to sanitary restrictions to perform a personal interview. It could also reach a more diverse population, despite geographical limitations. Participants were asked to answer the questionnaire regarding current and before the pandemic, using “retrospective post-then-pre” design, where information “during” and “before” pandemic were collected at the same time respondents were asked to answer at first the present information and then, the past information [20,21]. The post/pre-design was selected to offer a useful methodology for documenting the impact of the pandemic in everyday life and assess changes in EC over time, since a comparison of the pre-test scores with their posttest scores can result in inaccurate pre-test scores due to “response shift bias” [22].

The survey was composed of an initial page describing the aims and information on the ethics and the consent form. Participants were volunteers and did not receive any reward for their participation following the National Research Ethics Commission guidelines [23]. There was no time limit for completing the questionnaire. Individuals’ responses were registered after clicking on the “submit” button. Participants could stop answering the questionnaire at any step before the submission; in this case, the responses would not be registered. Incomplete questionnaires were removed from the final sample.

Researchers used the Brazilian version of the ecSI2.0™BR questionnaire, previously validated with the Brazilian adult population to assess EC [24] which was approved by an Ethics Committee (CAAE 24415819.2.0000.8101), according to Declaration of Helsinki guidelines. No names or other information that could identify the participant was requested.

The structured questionnaire included forty questions, divided into four different sections. Participants were asked to answer the questions regarding the period during and before the pandemic using the “retrospective post-then-pre” design. In this model, information regarding before and after are collected simultaneously, after the event or intervention has occurred [20]. At first, participants are asked to report their behavior or understanding as a result of the event (post) and, second, participants are asked to report their behavior before the event (pre) [21].

The first part of the questionnaire had sociodemographic data: age, gender, region of residence, level of education, income, employment situation, family income changes, and the number of individuals living at home, using questions from the Brazilian National Institute of Geography and Statistics (IBGE) as a reference [25].

The second part had anthropometrics and health information as weight (current and before pandemic), height, and whether the respondent or a family member tested positive for COVID-19.

In the third part, EC was accessed using the Brazilian Satter eating competence inventory (ecSI2.0™BR), composed of 16 questions and validated for online use among the Brazilian adult population [24]. The ecSI2.0™ is available at https://www.needscenter.org/ upon approval of an application [26]. The NEEDs Center previously approved the use of ecSI2.0™BR for this study. Respondents were asked to answer “current” and “before” confinement. A minimum total score of 32 is the cut-point to consider the individual as a competent eater, without established score cutoffs for each component [27,28].

The fourth part of the questionnaire investigated food consumption and eating habits changes during the pandemic period: frequency of consumption of fruits, vegetables, and artificial drinks (sugary beverages such as industrialized juice, tea, and soft drinks). For this purpose, it was selected some questions used in the Brazilian Food Questionnaire (VIGITEL) [29] that is already validated for online use [30]. Respondents were asked to answer “current” and “before” confinement. We included a question asking if the respondent noticed any change in the amount and the frequency of food eaten. In addition, we asked who is responsible for food preparation at home (if meals are ready-to-eat; if the respondent prepares meals; and if meals are prepared at home by another person).

The scores of ecSI2.0™BR and its four components were presented by means and standard deviation. The comparison of the scores before and during the pandemic was performed by paired Student’s t-test. The raw frequencies and percentages of respondents who showed eating competence (total EC score ≥ 32) were presented. The MCNemar test was used to compare these frequencies before and during the pandemic. Statistical analyses were performed using IBM SPSS Statistics, version 22. The tests took into consideration two-tailed hypotheses and a significance level of 5%.

## 3. Results

The study obtained 340 responses, but only responses from individuals aligned to the inclusion criteria and responded all the questions were considered, reducing the number of valid observations to 302 (88.8% of the total questionnaires). The sample consisted predominantly of women (*n* = 232; 76.82%), and mostly ≥ 40 years (Table 1). Most participants had a higher education level (54.6% graduate and 40.7% undergraduate), and most lived with other family members. During the pandemic, 66.5% (*n* = 201) of the respondents reported they did not reduce their income, and 42.5% did not change their job, but they were working from home.

In general, the EC differs before and during the pandemic considering its four components (eating attitude, food acceptance, internal regulation and contextual skills) and the total score for EC (*p* < 0.05). Considering gender, EC was lower for all components among females during the pandemic (*p* < 0.05) but did not differ among males. The EC (total) was lower during the pandemic compared to before it, regardless of age. However, contextual skills did not differ among the groups regardless of age and food acceptance among individuals up to 40 y/o.

Individuals with high school education did not differ EC (total and each component) during the pandemic. EC was lower among undergraduate and graduate individuals during the pandemic than before (*p* < 0.05), except for food acceptance and contextual skills among undergraduate and contextual skills among graduate individuals that did not differ.

Considering individuals who reduced the income during the pandemic, the EC lowered for all components and the total (*p* < 0.05), except for food acceptance that did not differ. However, the EC lowered during the pandemic regardless of income reduction (except for contextual skills among those with no income reduction, which did not differ) (Table 1).

EC lowered among individuals who had or had not COVID-19 (*p* < 0.05). However, it did not differ on contextual skills among those who did not have COVID-19 and food acceptance and contextual skills among those tainted by Sars-CoV-2. Individuals who had relatives tainted by Sars-CoV-2 presented lower EC during the pandemic than before (*p* < 0.05). Among those whose relatives were not infected, the EC did not differ.

Individuals who increased weight during the pandemic (21.8% of the sample) had a reduction in EC (total and components) (*p* < 0.05). The group that maintained weight during the pandemic (66.5% of the sample) did not change total EC, but showed a reduction in eating attitude and internal regulation (*p* < 0.05). EC did not differ among those who lost weight during the pandemic (11.5% of the sample).

Among those who reported a decrease in fruit and vegetable consumption, there was a reduction in total EC and all four components (*p* < 0.05). Among those who maintained fruit consumption, there was a reduction in total EC and internal regulation (*p* < 0.05). The group that maintained vegetable consumption showed a reduction in total EC and eating attitude and internal regulation (*p* < 0.05). The group that reported an increase in fruit consumption showed no changes in total EC and components, except for contextual skills, which increased. Those who increased vegetable consumption showed an increase in total EC and components (*p* < 0.05), except for internal regulation.

The group that reported a decrease in the consumption of industrialized beverages had no changes in total EC, eating attitude, and food acceptance but showed a reduction in internal regulation and increased contextual skills. The group that maintained the consumption of industrialized beverages showed a loss of total EC and components (*p* < 0.05), except for contextual skills, which remained the same. Among those who increased the consumption of industrialized beverages, there was a reduction in EC (total and all components) (*p* < 0.05).

EC (total and all components) lowered among individuals who increased food consumption during the pandemic period (*p* < 0.05). Among individuals that maintained or reduced food consumption, EC did not differ.

Individuals who buy ready-to-eat meals showed a reduction in total EC and all components (*p* < 0.05). There was no reduction in the contextual skills component among those who usually prepare their food. However, there was a reduction in the total EC and the other components (*p* < 0.05). Individuals with meals prepared at home by another person did not report differences in total EC, food acceptance, and contextual skills. However, they showed a decrease in eating attitude and internal regulation (*p* < 0.05).

## 4. Discussion

The impact of social isolation during the pandemic can vary by income, education, age, and gender [2]. However, the reduction in EC observed across the sample is alarming regardless of the variables evaluated. Considering the sample profile (mostly female, aged ≥ 40 years old, with a higher education level, and living with a family member), the outcomes reveal an isolation negative impact on EC that may reflect throughout the family and that should be the focus of public health strategies in the years that follow the pandemic.

Some aspects highlight the need to develop public policies capable of improving the situation. It is well known that higher educational level has been usually related with higher socioeconomic status, which has been associated with better diet quality [15]. In addition, a previous study with the Brazilian population showed that competent eaters (EC total score ≥ 32) are primarily individuals >40 years; graduated, and had a high income [31]. Despite the majority of this study’s sample having a profile similar to that of the aforementioned studies, the present study showed that, in general, EC (total score) decreased (31.60 ± 8.26 before pandemic vs. 29.00 ± 9.72 during pandemic *p* 0.000), revealing that restrictive measures during the pandemic play a negative impact on EC. The same has occurred analyzing the four components of EC, which have also decreased.

The data obtained appears to be a trend as a COVID-19 impact. Resembling results were published by Górnicka et al. [3], who investigated dietary changes among Polish adults during the pandemic and their associations with sociodemographic data and lifestyle changes. Although related to a sample of partially distinct characteristics, authors found that those >40 years old, having children, not working, and not consuming homemade meals might be more exposed to unhealthy behaviors [3].

The effect of the COVID-19 on the worldwide economy is significant and financial loss during quarantine is a difficult socioeconomic harm in Brazil [2]. The pandemic crisis might throw millions of workers into unemployment [32]. Since the pandemic generated an economic crisis, unemployment rose extensively, worsening health and social insecurity [33]. Our results showed that among individuals who reduced income during the pandemic (33.4%), all components and total EC lowered (*p* < 0.05), except for food acceptance. This finding is aligned with prior studies showing that low-income individuals have less favorable dietary patterns and show less EC [34,35].

Food insecurity is inversely associated with diet quality [36]. A recent study showed that females, with lower education and incomes, have higher overweight and obesity levels in Brazil. There is an association between unhealthy food intake and poverty [37]. The ability to be a competent eater is affected by food insecurity and/or income restrictions, as having worries about money for food is associated with a lack of EC [38,39]. Bezerra et al. (2020) investigated the perception of social isolation during the COVID-19 pandemic with a sample of 16.440 Brazilian respondents (69% females, mean age 41 y/o). Authors found that social interactivity was the most affected field among individuals with higher education and income, and financial problems generated a more significant impact among those with low income and education [2]. Considering that part of society has experienced a reduction of their income, it is expected to reflect their consumption patterns. On the other hand, those who have not changed their income may have changed to convenience foods when purchasing or cooking their meals [32].

Regarding health data, individuals who had relatives infected by Sars-CoV-2 had a decrease in EC during the pandemic, probably due to the stress about the outcome of the infection since stress can be associated with negative changes in eating behavior [40,41]. Adding to the pandemic scenario as a whole, these changes can be leveraged. Therefore, the decrease in EC can be a consequence of the experience of individuals with relatives affected by the virus.

The group that maintained weight during the pandemic (66.5% of the sample) did not change total EC, but showed a reduction in eating attitude and internal regulation (*p* < 0.05). EC did not differ among those who lost weight during the pandemic (11.5% of the sample). The association between weight and EC is well documented in studies that found an inverse association of EC and weight [8,38,42,43,44,45]. It must be highlighted that obesity was one of the worse global health problems even before the COVID-19 pandemic and it is a risk factor for other metabolic diseases [13]. Moreover, it is known that individuals with obesity and metabolic disorders have a higher risk of developing critical COVID-19 outcome [12]. Thus, the adequate control of weight and metabolic complications is vital to reduce the risk of severe forms of COVID-19.

Energy balance primarily defines weight gain or loss [46]. However, some foods are directly associated with energy supply, which can affect body weight. The consumption of fruits and vegetables has a positive effect in this regard [47], while sugary beverages can contribute to weight gain by increasing caloric intake [48]. As most of the sample did not report weight change, the majority’s consumption of these foods was also not changed.

In addition, fruit and vegetable intake (FV) is essential to ensure micronutrients that can boost immune function [13]. FV consumption is used as an indicator of health, and it is adequate when consumed at least five times per week [47]. In our study, EC (total and all four components) decreased among those who reported decreased FV consumption during the pandemic. On the contrary, EC did not change among those who reported an increase in FV consumption, showing an increase in the contextual skills component. Those who increased vegetable consumption showed an increase in total EC and components (*p* < 0.05), except for internal regulation which did not differ. Similar results were reported by Queiroz et al. (2020), who found a positive association between EC and FV consumption among Brazilian adults [31]. These results are consistent with prior studies in which EC was related to diet quality [34,35,42,49]. Thus, considering that the pandemic negatively affects EC, a worsening in food quality is expected. In Brazil, Malta et al. (2020) noticed a decrease in the frequency of consumption of healthy foods during the pandemic, and the most significant decrease was observed in the regular consumption of vegetables [16]. The decrease in FV consumption in the pandemic leads to a deficiency in micronutrients, which can harm the immune function, increasing susceptibility to infection [50].

It is also important to consider problems related to food distribution and the changes in global supply chains, which have adverse effects like the lack of some fresh food supplies [32]. The dominance of the food industry and supermarket chains and the reduction in the activities of the local markets put the local food producer at a disadvantage compared to the supply of processed foods [16,17,32]. Furthermore, it can explain the decrease in the consumption of fresh food like FV. In Brazil, Ribeiro-Silva et al. (2020) analyzed the effects of pandemic on food and nutrition security. They highlighted losses in the supply of fresh food from family farming, especially FV, and reduced consumption of fresh foods, increasing weight gain or eating disorders associated with physical inactivity and social distancing [51].

The regular intake of soft drinks is a mark of unhealthy eating habits. Soft drinks consumption is connected with higher energy intake and glycemic index, lower intake of fruits and fiber, and higher intake of fast foods [48]. In our study, those who increased their consumption of industrialized beverages showed a reduction in total EC and all components. It is essential to highlight that sugar intake improves serotonin production, positively affecting mood momentarily and transiently, encouraging people to consume foods with sugar frequently [13]. This unhealthy nutritional habit could increase the risk of developing obesity. The group that reported a decrease in the consumption of industrialized beverages had no changes in total EC, eating attitude, and food acceptance but showed a reduction in internal regulation and increased contextual skills. Those who maintained the consumption of industrialized beverages showed a loss of total EC and components, except for contextual skills, which remained the same. Similar results were reported by Queiroz et al. (2020), who found an inverse association between EC and artificial juice or soda consumption among Brazilian adults [31].

Restrictive measures conduct to irregular eating patterns and frequent snacking, associated with higher caloric ingestion and risk of excess weight [12,41]. Since quarantine is associated with the interruption of the routine, this could result in boredom and stress, associated with a greater energy intake [13,36,41]. In our sample, individuals who increased food consumption showed decreased EC (total and all components). On the contrary, individuals that maintained or reduced food consumption showed no difference in EC. Previous studies investigating dietary practices during pandemics showed similar results. Ammar et al. [6] conducted an online study in April 2020, in seven languages, to investigate the behavioral and lifestyle consequences of COVID-19 restrictions. Authors found an increase in unhealthy diet during COVID-19, with food consumption and meal patterns (the type of food, eating out of control, snacks between meals, number of main meals) more unhealthy in the period of the pandemic [6]. Górnicka et al. (2020) also found similar results among Polish adults, who showed increased food consumption and snacking during restrictive measures, especially among those with higher BMI [3]. Similar results were noticed by Cosgrove and Wharton (2021), who found that participants most often reported purchasing more food in various food categories [36]. In France, the nutritional quality of the diet was also higher before the lockdown than during the pandemic [10].

On the contrary, Rodríguez-Pérez et al. observed an improvement in Spanish adults’ dietary behaviors during the COVID-19 restrictions, like a higher intake of FV compared to their habits [15]. Lohse et al. (2010) showed that, among elderly Spanish, EC was positively associated with greater adherence to a Mediterranean diet, which is considered a healthy eating pattern [52]. Cosgrove and Wharton (2021) also noticed positive changes among US adults since participants reported improvements in dietary healthfulness during the pandemic [36]. There are some possible explanations for these findings: the greater possibility of preparing a meal at home, more free time to take care of health during home confinement, or food choice motives. For example, a French study with 938 adults revealed that improved diet quality was associated with an increase in the importance of weight control. Reduced diet quality was associated with the increased importance of mood, signing that food choice motives are essential factors related to changes in diet quality [10].

Directly staying at home affects lifestyle, including dietary habits and eating patterns [4,5]. Cosgrove and Wharton (2021), in an online survey with 958 US respondents, found changes concerning food shopping from grocery stores, farmer’s markets, sit-down restaurants, and fast-food restaurants. All have decreased and increases were observed in online grocery shopping and ordering takeout [36]. Usually, a turbulent lifestyle and less time in everyday life are barriers to incorporating a healthy diet in daily life. It was expected that, while staying at home, individuals should have had more time to cook and organize their meals [15]. However, individuals who are not used to managing the food context may have found it more difficult when faced with this new reality. In fact, in our study, individuals who buy ready-to-eat meals showed a reduction in total EC and all components. There was no reduction in the contextual skills component among those who usually prepare their food, which is expected to be higher among individuals that usually cook [7]. Individuals with meals prepared at home by another person did not report differences in total EC, food acceptance, and contextual skills. However, they showed a decrease in eating attitude and internal regulation, which are not related to managing the food context. This is probably because worries about food, whether for aesthetic reasons or health reasons, favor negative eating attitudes [53].

Although home cooking might have increased, it is not clear whether quarantine and social isolation have improved the nutrition quality of dishes. Individuals of different ages and lifestyle situations need skills in food purchasing and preparation to maintain or achieve a healthy lifestyle [54]. Therefore, the contextual skill component seems to be an important aspect that would help to deal with eating in this pandemic scenario since it is linked to the ability to plan meals, develop food purchasing strategies, and have culinary skills that enable food autonomy, managing the time dedicated to preparing and consuming meals [7,38].

A previous study showed that the contextual skills component has a positive association among the Brazilian adult population with age, education level, income, BMI, and quality of food consumption [31]. There is some evidence that improving Contextual Skills in weight management interventions helps weight loss (Lohse et al., 2018). The pandemic shows the necessity for nutrition and food preparation education programs to deal with feeding in everyday life and during an emergency [3].

Cosgrove (2021) noted that most studies assessing COVID-19-related changes in lifestyle behaviors have focused on physical activity and sedentary behavior, neglecting the influence of diet during this health crisis [36]. This study is the first to investigate changes in EC during the COVID-19 pandemic, making the comparison with other results more challenging. Although the questionnaire was directed to all Brazilian regions and different segments of the adult population, the main limitation of this study is the convenience sample. Our sample had a higher proportion of female respondents, limiting the generalizability of the results to the Brazilian population. The socioeconomic level of the sample is also a potential bias. The pandemic does not uniformly affect all people, and the highest number of deaths is usually observed among the most impoverished populations because they are more likely to have chronic conditions that put them at a higher risk of mortality [33].

Individuals with low access to information are more predisposed to ignore government health warnings since they are more likely to be affected by misinformation and miscommunication [33]. The present research was carried out one year after the beginning of the containment measures, during the worst period of the second wave of the pandemic in Brazil. There was limited opportunity to conduct studies involving respondents. Individuals’ adherence to isolation may have been reduced over time [18], and, at present, the measures to counter the pandemic have already led to changes in purchasing and consumption habits [32]. A great discrepancy was observed among individuals with higher income and education (the majority) and individuals with lower income and education (the minority). Thus, this work does not represent the EC of the whole Brazilian population during the pandemic but only of the sample studied.

The COVID-19 pandemic threatens the health of individuals and affects social and economic well-being around the world, revealing the fragility of the global supply chains and the new challenges that local administrations have to face. In addition to the obligation to combat the increase in obesity and malnutrition, there is a tendency for new problems, which will require greater attention [32]. The COVID-19 pandemic imposed new challenges to support a healthy diet [50]. In this context, the challenge of public policies is to promote and ensure food and nutrition security after the social and health crisis created by the pandemic, which involves an effort in planning the development of distribution of food based on international health standards, but focusing on the preservation of food production and consumption’s local culture, knowledge, and traditions [2,32,50].

The context of the pandemic may allow to introduce new questions on the feeding habits and patterns [32]. It is time to think about the relationship between food and eating. EC is an indicator of positive eating-related behavior, is associated with health-promoting food consumption and health outcomes [7,38,55]. Thus, more than ever, the time seems to be favorable to invest in improvements in EC since it involves eating attitudes and behaviors, internal regulation of intake, food acceptance, and skills related to selecting, preparing, and planning meals.

The present study’s strength is that we had responses from all Brazilian regions. In addition, this is an unprecedented study that draws attention to the aspect of EC changes caused by the pandemic. However, our study presents some limitations as the non-representativeness of the sample and the results cannot be generalized to the entire adult population since we used a convenience sample mainly composed of females. Finally, the duration of the research could influence the results due to the very dynamic feature of the COVID-19. Another potential study bias is the internet survey dissemination. However, during the pandemic period, the internet is the primary way to achieve participants.

## 5. Conclusions

Eating competence, is an indicator of positive eating-related behavior, associated with health-promoting food consumption. In the studied sample, our hypothesis was confirmed in which total EC score lowered during the pandemic, and the decrease was worst among those who reported weight gain, decreased the consumption of fruit and vegetables, and increased the consumption of sugary beverages, after the beginning of the pandemic. The contextual skill component seems relevant in this scenario, where our life and routines were changed entirely, demonstrating that the ability to manage the food context is essential, primarily when sanitary and economic situations represent a new challenge. Further studies are necessary to evaluate EC after the pandemic, aiming to focus on recommendations for lifestyle modifications after this challenging time.

## Figures and Tables

**Table 1:** Sub-scores of the ecSI2.0™BR scale categorized by variables before and during the COVID-19 pandemic (*n* = 302, Brazil).

	Eating Attitude	Food Acceptance	Internal Regulation	Contextual Skills	Total	ecSI2.0™BR ≥ 32
	Mean (SD)	Mean (SD)	Mean (SD)	Mean (SD)	Mean (SD)	Freq. (%)
General (*n* = 302)						
Before pandemic	12.68 (3.74)	5.18 (2.47)	4.10 (1.43)	9.73 (3.31)	31.69 (8.26)	160 (53.0%)
During pandemic	11.77 (4.28)	4.95 (2.53)	3.74 (1.68)	9.53 (3.61)	29.99 (9.72)	143 (47.4%)
*p*	0.000 *	0.001 *	0.000 *	0.293 *	0.000 *	0.019 **
Gender *						
Female (*n* = 232)						
Before pandemic	12.62 (3.70)	5.40 (2.41)	3.97 (1.38)	10.06 (2.99)	32.04 (7.84)	128 (55.2%)
During pandemic	11.65 (4.00)	5.10 (2.47)	3.56 (1.66)	9.62 (3.56)	29.92 (9.30)	106 (45.7%)
*p*	0.000 *	0.000 *	0.000 *	0.032 *	0.000 *	0.000 **
Male (*n* = 70)						
Before pandemic	12.89 (3.92)	4.46 (2.56)	4.53 (1.52)	8.64 (4.01)	30.51 (9.48)	32 (45.7%)
During pandemic	12.17 (5.13)	4.47 (2.67)	4.34 (1.61)	9.24 (3.80)	30.23 (11.08)	37 (52.9%)
*p*	0.060 *	0.908 *	0.102 *	0.184 *	0.740 *	0.277 **
Age						
Up to 40 years (*n* = 145)						
Before pandemic	12.39 (4.03)	5.31 (2.61)	3.99 (1.47)	9.23 (3.49)	30.92 (9.00)	74 (51.0%)
During pandemic	11.32 (4.28)	5.09 (2.66)	3.55 (1.71)	8.93 (3.77)	28.89 (9.96)	61 (42.1%)
*p*	0.000 *	0.052 *	0.000 *	0.320 *	0.002 *	0.019 **
More than 40 years (*n* = 157)						
Before pandemic	12.95 (3.45)	5.06 (2.35)	4.2 (1.39)	10.19 (3.07)	32.39 (7.47)	86 (54.8%)
During pandemic	12.18 (4.26)	4.83 (2.4)	3.91 (1.63)	10.09 (3.38)	31.01 (9.42)	82 (52.2%)
*p*	0.000 *	0.006 *	0.000 *	0.659 *	0.003 *	0.503 **
Schooling level **						
High School (*n* = 14)						
Before pandemic	13.29 (4.18)	5.14 (2.68)	4.57 (1.45)	10.50 (3.74)	33.50 (9.34)	9 (64.3%)
During pandemic	12.43 (4.70)	5.21 (2.22)	4.07 (1.69)	9.43 (4.38)	31.14 (10.17)	8 (57.1%)
*p*	0.201 *	0.818 *	0.187 *	0.059 *	0.164 *	1.000 **
Undergraduate (*n* = 123)						
Before pandemic	13.02 (3.49)	5.08 (2.46)	4.32 (1.37)	9.27 (3.55)	31.68 (8.04)	61 (49.6%)
During pandemic	12.13 (4.23)	4.89 (2.52)	3.93 (1.74)	9.25 (3.75)	30.20 (9.88)	60 (48.8%)
*p*	0.001 *	0.122 *	0.000 *	0.964 *	0.038 *	1.000 **
Graduate (*n* = 165)						
Before pandemic	12.38 (3.88)	5.25 (2.48)	3.90 (1.45)	10.01 (3.05)	31.54 (8.36)	90 (54.5%)
During pandemic	11.44 (4.29)	4.98 (2.57)	3.56 (1.62)	9.75 (3.45)	29.74 (9.62)	75 (45.5%)
*p*	0.000 *	0.001 *	0.000 *	0.231 *	0.000 *	0.001 **
Family Income Changes						
Did not reduce (*n* = 201)						
Before pandemic	12.75 (3.68)	5.12 (2.43)	4.24 (1.40)	9.72 (3.18)	31.84 (7.77)	103 (51.2%)
During pandemic	12.00 (4.22)	4.91 (2.50)	3.93 (1.68)	9.77 (3.52)	30.60 (9.38)	95 (47.3%)
*p*	0.000 *	0.006 *	0.000 *	0.849 *	0.008 *	0.229 **
Reduced (*n* = 101)						
Before pandemic	12.54 (3.89)	5.29 (2.57)	3.81 (1.45)	9.75 (3.55)	31.4 (9.20)	57 (56.4%)
During pandemic	11.32 (4.39)	5.04 (2.60)	3.36 (1.61)	9.07 (3.76)	28.78 (10.30)	48 (47.5%)
*p*	0.000 *	0.076 *	0.000 *	0.032 *	0.000 *	0.022 **
COVID-19 infection						
No (*n* = 280)						
Before pandemic	12.54 (3.76)	5.13 (2.43)	4.09 (1.44)	9.74 (3.33)	31.50 (8.34)	148 (52.9%)
During pandemic	11.74 (4.22)	4.94 (2.50)	3.76 (1.67)	9.62 (3.56)	30.06 (9.65)	133 (47.5%)
*p*	0.000 *	0.006 *	0.000 *	0.534 *	0.000 *	0.032 **
Yes (*n* = 22)						
Before pandemic	14.45 (3.07)	5.77 (2.93)	4.18 (1.33)	9.68 (3.01)	34.09 (6.90)	12 (54.5%)
During pandemic	12.09 (5.11)	5.09 (2.96)	3.50 (1.77)	8.45 (4.19)	29.14 (10.85)	10 (45.5%)
*p*	0.016 *	0.065 *	0.032 *	0.210 *	0.041 *	0.625 **
COVID-19 infection in a family member						
No (*n* = 158)						
Before pandemic	12.39 (3.68)	5.15 (2.35)	3.94 (1.43)	9.54 (3.51)	31.03 (8.36)	81 (51.3%)
During pandemic	11.83 (4.16)	5.03 (2.46)	3.79 (1.60)	9.71 (3.75)	30.36 (9.77)	78 (49.4%)
*p*	0.002 *	0.200 *	0.059 *	0.508 *	0.173 *	0.701 **
Yes (does not live with me *n* = 113)						
Before pandemic	12.65 (3.85)	4.91 (2.47)	4.31 (1.39)	9.85 (3.19)	31.72 (8.25)	58 (51.3%)
During pandemic	11.49 (4.49)	4.58 (2.40)	3.69 (1.77)	9.12 (3.53)	28.88 (9.80)	48 (42.5%)
*p*	0.000 *	0.011 *	0.000 *	0.032 *	0.000 *	0.013 **
Yes (live with me *n* = 31)						
Before pandemic	14.26 (3.42)	6.32 (2.84)	4.13 (1.52)	10.26 (2.56)	34.97 (7.14)	21 (67.7%)
During pandemic	12.48 (4.14)	5.90 (3.08)	3.65 (1.78)	10.13 (3.14)	32.16 (8.96)	17 (54.8%)
*p*	0.007 *	0.025 *	0.001 *	0.805 *	0.026 *	0.219 **
Weight changes						
Decreased (*n* = 35)						
Before pandemic	11.77 (4.66)	5.91 (2.23)	3.86 (1.73)	9.46 (4.10)	31.00 (10.48)	21 (60.0%)
During pandemic	11.77 (5.12)	5.63 (2.53)	4.11 (1.43)	10.03 (3.69)	31.54 (9.75)	18 (51.4%)
*p*	1.000 *	0.185 *	0.263 *	0.442 *	0.702 *	0.453 **
Maintained (*n* = 201)						
Before pandemic	13.81 (3.40)	5.17 (2.43)	4.10 (1.40)	9.84 (3.12)	32.42 (7.56)	110 (54.7%)
During pandemic	12.62 (3.93)	5.14 (2.45)	3.93 (1.52)	10.11 (3.25)	31.81 (8.63)	107 (53.2%)
*p*	0.000 *	0.597 *	0.000 *	0.137 *	0.069 *	0.690 **
Increased (*n* = 66)						
Before pandemic	11.24 (3.77)	4.80 (2.68)	4.21 (1.34)	9.56 (3.43)	29.82 (8.78)	29 (43.9%)
During pandemic	9.18 (4.22)	4.02 (2.55)	2.95 (2.00)	7.50 (3.95)	23.65 (10.31)	18 (27.3%)
*p*	0.000 *	0.001 *	0.000 *	0.000 *	0.000 *	0.007 **
Fruit consumption						
Decreased (*n* = 62)						
Before pandemic	12.19 (3.43)	4.76 (2.17)	4.23 (1.38)	9.92 (3.36)	31.10 (7.02)	31 (50.0%)
During pandemic	10.58 (4.53)	3.81 (2.13)	3.10 (1.91)	8.35 (4.15)	25.84 (10.84)	20 (32.3%)
*p*	0.000 *	0.000 *	0.000 *	0.024 *	0.000 *	0.052 **
Maintained (*n* = 195)						
Before pandemic	12.95 (3.79)	5.30 (2.53)	4.11 (1.45)	10.02 (3.26)	32.37 (8.56)	109 (55.9%)
During pandemic	12.10 (4.31)	5.21 (2.49)	3.91 (1.60)	9.89 (3.45)	31.11 (9.33)	98 (50.3%)
*p*	0.000 *	0.129 *	0.001 *	0.447 *	0.000 *	0.007 **
Increased (*n* = 45)						
Before pandemic	12.16 (3.92)	5.24 (2.60)	3.89 (1.40)	8.24 (3.07)	29.53 (8.26)	20 (44.4%)
During pandemic	11.98 (3.58)	5.42 (2.80)	3.87 (1.46)	9.60 (3.26)	30.87 (8.34)	25 (55.6%)
*p*	0.646 *	0.263 *	0.888 *	0.001 *	0.113 *	0.063 **
Vegetable consumption						
Decreased (*n* = 63)						
Before pandemic	11.54 (3.75)	4.92 (2.50)	3.83 (1.28)	9.78 (2.79)	30.06 (8.07)	26 (41.3%)
During pandemic	9.22 (4.01)	3.68 (2.37)	2.60 (1.62)	6.63 (3.40)	22.14 (9.22)	9 (14.3%)
*p*	0.000 *	0.000 *	0.000 *	0.000 *	0.000 *	0.000 **
Maintained (*n* = 202)						
Before pandemic	13.18 (3.67)	5.42 (2.48)	4.17 (1.45)	10.31 (3.19)	33.08 (8.02)	125 (61.9%)
During pandemic	12.40 (4.02)	5.35 (2.47)	4.03 (1.55)	10.41 (3.17)	32.18 (8.55)	113 (55.9%)
*p*	0.000 *	0.131 *	0.019 *	0.523 *	0.003 *	0.008 **
Increased (*n* = 37)						
Before pandemic	11.86 (3.65)	4.32 (2.24)	4.16 (1.54)	6.49 (2.94)	26.84 (7.71)	9 (24.3%)
During pandemic	12.65 (4.60)	4.97 (2.46)	4.08 (1.66)	9.70 (3.76)	31.41 (9.94)	21 (56.8%)
*p*	0.051 *	0.001 *	0.446 *	0.000 *	0.000 *	0.000 **
Sugary beverages						
Decreased (*n* = 39)						
Before pandemic	11.18 (3.93)	4.18 (2.16)	4.08 (1.51)	6.56 (3.28)	26.00 (7.90)	9 (23.1%)
During pandemic	10.62 (5.42)	4.56 (2.35)	3.49 (1.75)	9.10 (3.32)	27.77 (10.60)	16 (41.0%)
*p*	0.404 *	0.168 *	0.016 *	0.003 *	0.309 *	0.092 **
Maintained (*n* = 210)						
Before pandemic	13.24 (3.48)	5.44 (2.42)	4.20 (1.43)	10.46 (2.97)	33.34 (7.68)	128 (61.0%)
During pandemic	12.57 (3.65)	5.22 (2.48)	3.97 (1.62)	10.27 (3.33)	32.03 (8.73)	117 (55.7%)
*p*	0.000 *	0.000 *	0.001 *	0.236 *	0.000 *	0.027 **
Increased (*n* = 53)						
Before pandemic	11.55 (4.10)	4.89 (2.72)	3.72 (1.34)	9.19 (3.16)	29.34 (8.45)	23 (43.4%)
During pandemic	9.43 (4.70)	4.19 (2.70)	3.00 (1.65)	6.92 (3.70)	23.55 (9.80)	10 (18.9%)
*p*	0.000 *	0.006 *	0.001 *	0.000 *	0.000 *	0.000 **
Amount of food						
Reduced (*n* = 56)						
Before pandemic	13.02 (3.96)	5.36 (2.45)	4.34 (1.62)	9.23 (4.15)	31.95 (8.98)	30 (53.6%)
During pandemic	12.86 (4.37)	5.34 (2.34)	4.14 (1.47)	9.80 (3.83)	32.14 (9.46)	33 (58.9%)
*p*	0.747 *	0.914 *	0.213 *	0.362 *	0.875 *	0.648 **
Maintained (*n* = 99)						
Before pandemic	14.02 (3.14)	5.38 (2.38)	4.33 (1.34)	10.67 (2.91)	34.40 (7.45)	63 (63.6%)
During pandemic	13.76 (3.20)	5.36 (2.40)	4.27 (1.39)	10.85 (3.01)	34.24 (7.79)	63 (63.6%)
*p*	0.015 *	0.754 *	0.158 *	0.268 *	0.539 *	1.000 **
Increased (*n* = 147)						
Before pandemic	11.65 (3.75)	4.97 (2.55)	3.85 (1.38)	9.29 (3.07)	29.76 (8.02)	67 (45.6%)
During pandemic	10.01 (4.19)	4.53 (2.63)	3.22 (1.78)	8.54 (3.62)	26.31 (9.63)	47 (32.0%)
*p*	0.000 *	0.000 *	0.000 *	0.009 *	0.009 *	0.000 **
Meals during pandemic						
Bought ready-to-eat (*n* = 24)						
Before pandemic	11.54 (3.99)	3.88 (2.36)	3.67 (1.74)	8.29 (3.41)	27.38 (9.04)	10 (41.7%)
During pandemic	9.21 (4.64)	3.17 (2.28)	2.71 (1.76)	6.75 (3.31)	21.83 (9.76)	4 (16.7%)
*p*	0.001 *	0.023 *	0.005 *	0.015 *	0.001 *	0.031 **
Prepared by myself (*n* = 135)						
Before pandemic	12.93 (3.81)	5.83 (2.39)	4.18 (1.43)	10.51 (3.2)	33.44 (8.39)	85 (63.0%)
During pandemic	12.01 (4.22)	5.47 (2.48)	3.76 (1.74)	10.10 (3.76)	31.33 (10.00)	71 (52.6%)
*p*	0.000 *	0.003 *	0.000 *	0.157 *	0.001 *	0.003 **
Prepared at home by others (*n* = 143)						
Before pandemic	12.64 (3.63)	4.78 (2.42)	4.10 (1.37)	9.24 (3.23)	30.76 (7.62)	65 (45.5%)
During pandemic	11.97 (4.17)	4.77 (2.47)	3.90 (1.55)	9.47 (3.31)	30.10 (8.81)	68 (47.6%)
*p*	0.001 *	0.852 *	0.002 *	0.388 *	0.191 *	0.664 **

* Paired Student’s *t*-test, comparing before and during pandemic. ** McNemar test.

## Data Availability

The study did not report any data.
